# Calmodulin confers calcium sensitivity to the stability of the distal intracellular assembly domain of Kv7.2 channels

**DOI:** 10.1038/s41598-017-13811-4

**Published:** 2017-10-18

**Authors:** Alessandro Alaimo, Eider Nuñez, Paloma Aivar, Juncal Fernández-Orth, Carolina Gomis-Perez, Ganeko Bernardo-Seisdedos, Covadonga Malo, Alvaro Villarroel

**Affiliations:** Instituto Biofisika, Consejo Superior de Investigaciones Científicas, CSIC, UPV/EHU, Barrio Sarriena s/n, 48940 Leioa, Spain

## Abstract

Tetrameric coiled-coil structures are present in many ion channels, often adjacent to a calmodulin (CaM) binding site, although the relationship between the two is not completely understood. Here we examine the dynamic properties of the ABCD domain located in the intracellular C-terminus of tetrameric, voltage-dependent, potassium selective Kv7.2 channels. This domain encompasses the CaM binding site formed by helices A and B, followed by helix C, which is linked to the helix D coiled-coil. The data reveals that helix D stabilizes CaM binding, promoting *trans*-binding (CaM embracing neighboring subunits), and they suggest that the ABCD domain can be exchanged between subunits of the tetramer. Exchange is faster when mutations in AB weaken the CaM interaction. The exchange of ABCD domains is slower in the presence of Ca^2+^, indicating that CaM stabilization of the tetrameric assembly is enhanced when loaded with this cation. Our observations are consistent with a model that involves a dynamic mechanism of helix D assembly, which supports reciprocal allosteric coupling between the A-B module and the coiled-coil formed by the helix D. Thus, formation of the distal helix D tetramer influences CaM binding and CaM-dependent Kv7.2 properties, whereas reciprocally, CaM and Ca^2+^ influence the dynamic behavior of the helix D coiled-coil.

## Introduction

Coiled-coils are bundles of intertwined α-helices, a conformation that is one of the most widespread and versatile protein-protein interaction domains found in nature^1–3^. These domains are found in a subset of tetrameric ion channels^4,5^, and there is much evidence that these structures determine the stability and selectivity of multimerization^4,6^. Voltage-gated K^+^ channels are tetramers of α-subunits that constitute a K^+^-selective pore^7^. Two types of domains are involved in K^+^ channel assembly. In Shaker-related K^+^ channels, a cytoplasmic N-terminal domain (T1) is important for subfamily-specific channel assembly^8–10^. By contrast, C-terminal coiled-coil domains are required to assemble functional eag/erg, inward rectifier IRK1/Kir2.1 and Kv7 channels^4,11–15^. Coiled-coil domains have also been attributed key roles in the assembly of TRP^16^ and cyclic nucleotide-gated ion channels^17^. These structures are also found in SK K^+^ channels and in plant inward rectifying AKT1/KAT1 K^+^ channels^18–21^, among others.

The high-resolution structure of helix D in Kv7.4 shows this domain to be a self-assembling, parallel, four-stranded coiled-coil^6^. In accordance with the conserved sequence among Kv7 channel subtypes, crystallography studies of Kv7.1 revealed that its helix D also adopts a tetrameric, parallel-orientated, coiled-coil quaternary structure^5^. Pathological mutations located in helix D compromise the ability of Kv7.1 to reach the plasma membrane^22–24^. Moreover, a mutation underlying a form of long QT syndrome interferes in the interaction of the Kv7.1 helix D with the scaffold protein yotiao, which anchors protein kinase A and protein phosphatase 1 to the channel complex^25^. Furthermore, a mutation that disrupts coiled-coil formation in Kv7.2 channels leads to familiar epilepsy^26^. Thus, although the coiled-coil formed by helix D is not essential for Kv7 function and it can be replaced by an unrelated tetrameric coiled-coil domain, channel processing, surface expression and gating are affected by alterations to this helix^23,27–29^.

In addition to coiled-coil domains, many channels contain separate calmodulin (CaM) binding domains, including the SK, IK, TRP, Kv7 (KCNQ) and CNG channels. One feature of Kv7 channels is the absolute requirement of PIP_2_ binding to be functional and mounting evidence reveals that sensitivity to PIP_2_ is modulated by CaM binding^30,31^. Recent findings indicate that the function of helix D goes beyond providing a physical platform for channel assembly. Accordingly, the relationship between the coiled-coil and CaM binding domains has been examined recently in Kv7.2 channels, suggesting that the stability of the helix D coiled-coil affects CaM binding and that it results in altered PIP_2_ sensitivity^32^. Here, we investigated the contribution of helix D to CaM binding. We present evidence supporting the notion that the coiled-coil formed by helix D distal to the AB CaM-binding module indirectly favors *trans*-binding of CaM to Kv7.2, and that CaM binding stabilizes the tetrameric C-terminal assembly. Remarkably, it appears that the tetrameric assembly is sensitive to Ca^2+^, becoming more stable in the presence of this cation. Thus, our data reveal an important reciprocal crosstalk between CaM-binding and the subunit-recognition domains of Kv7 channels.

## Results and Discussion

**Figure 1:**
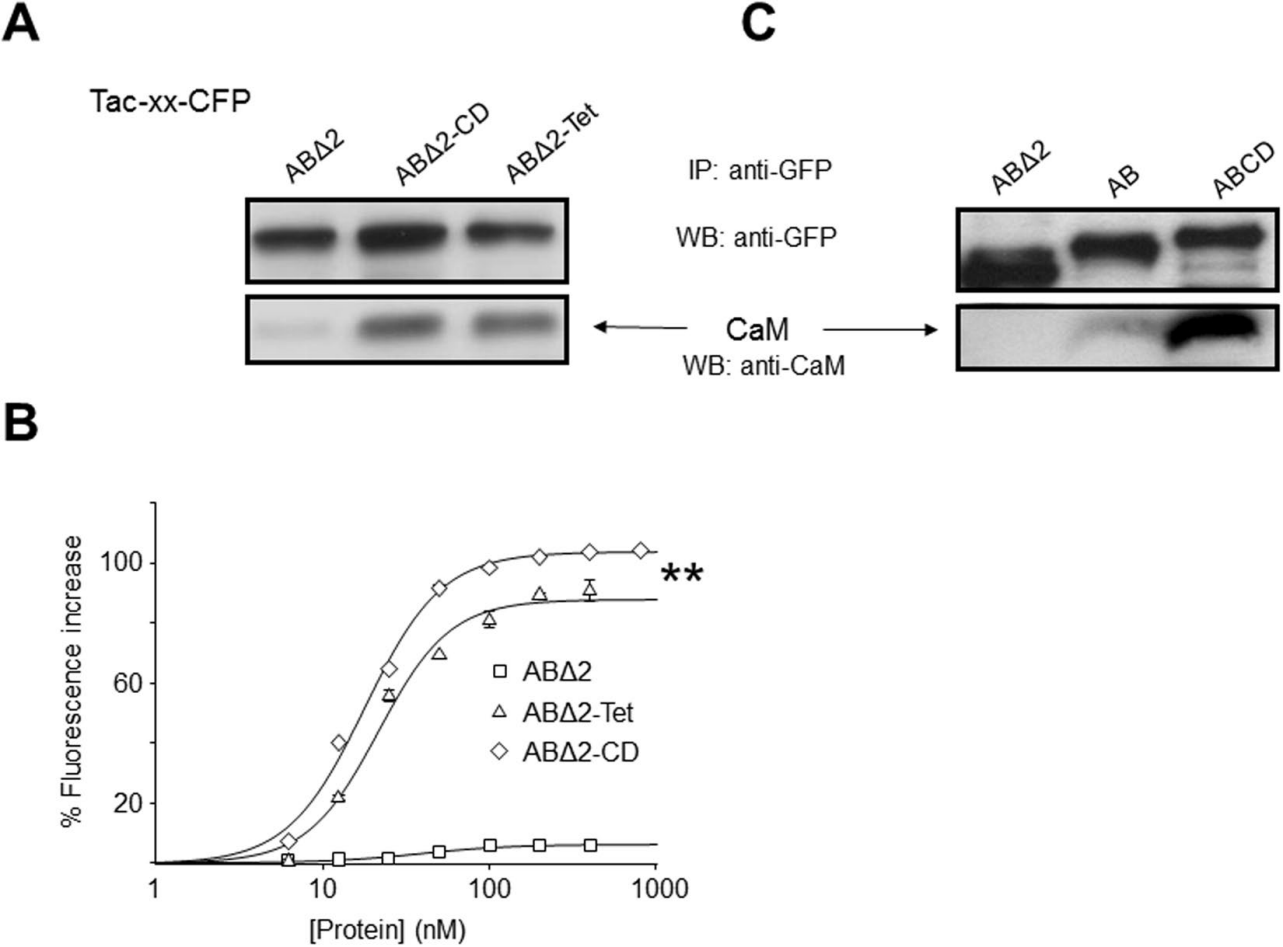
The presence of a tetramerization domain favors calmodulin binding to a membrane protein. (**A** and **C**) Tac-Kv7.2-CFP chimeras extracted from HEK293T cells were immunoprecipitated using anti-GFP antibodies, separated by SDS-PAGE, transferred to membranes (WB), and probed with anti-GFP and anti-CaM antibodies (n ≥ 3, see supplemental Figs 2 and 3 for the full blots). The bottom panel in (**C**) was obtained after a longer exposure than the top panel (see supplemental Fig. 3). (**B**) D-CaM (12.5 nM) fluorescence enhancement in the absence of Ca^2+^ (10 mM EGTA) at the indicated recombinant GST-fusion protein concentrations. The lines are the result of fitting a Hill equation to the data. The EC_50_ (nM) was 17.8 ± 1.1 and 21.5 ± 0.5, for ABΔ2-CD and ABΔ2-Tet, respectively. The asterisks indicate significantly different maximal fluorescence: **p < 0.01. The data represent the means ± SEM from 3 independent experiments.

To study how the assembly/tetramerization domain (CD module) of the Kv7.2 subunit influences CaM binding (see supplemental Fig. 1), we constructed chimeric proteins of the monomeric single transmembrane protein Tac and the Kv7.2 AB CaM-binding domain, and we expressed them in HEK293T cells^33^. The AB module carried a deletion in the A-B linker (denoted ABΔ2) that is compatible with Kv7.2 channel function^34^, and that improved protein yield and solubility when this domain was produced as a recombinant protein in bacteria (not shown). The chimera incorporated a GFP tag at the C-terminus that allowed the complexes to be pulled-down using anti-GFP antibodies. The signal from the CaM pulled-down, revealed with anti-CaM antibodies, was compared between the chimeras that incorporated the ABΔ2 and ABΔ2-CD modules. Furthermore, in a third chimera the CD module was replaced with an artificial, unrelated amino acid sequence (Tet) that adopts a tetrameric coiled-coil configuration^35^. Figure 1A shows a dramatic increase in the CaM signal when chimeras that incorporated either the natural or the artificial tetramerization signal were pulled-down. Thus, coiled-coil formation distal to the binding domain favors CaM engagement.

To gain further insight into the influence of a distal tetramerization signal on the interaction between the CaM and AB site, we produced GST-fused proteins in bacteria in the absence of CaM. As reported previously, most of the AB domain ends up in inclusion bodies when produced in the absence of CaM^36^. However, the protein from these inclusion bodies was denatured with urea, refolded and purified, recovering soluble monodisperse GST-tagged protein^37^ (Supplemental Fig. 4). This protein could be subjected to binding analyses using D-CaM, a dansylated derivate that reports conformational changes of CaM as an increase in fluorescence emission upon binding to a target protein^38–40^. Strikingly, D-CaM reported a very small increase in fluorescence when tested for its interaction with ABΔ2, whereas there was a robust increase in D-CaM fluorescence in the binding assay with a chimeric construct containing a distal artificial tetramerization sequence (ABΔ2-tet, Fig. 1B). The increase in D-CaM fluorescence was even greater when the natural assembly domain was present (ABΔ2-CD, Fig. 1B). The signal from ABΔ2 was too small to obtain reliable data on affinity, so we tested the impact of helices CD on CaM binding to helices AB with the full linker. Figure 1C compares the CaM signal after the pull-down of Tac chimeras expressed in HEK293T. The CaM signal from the Tac-ABΔ2 chimera was weaker than that from the Tac-AB chimera, suggesting that the linker helps stabilize CaM binding, an issue that was not explored further in this report. Importantly, the CaM signal after pull-down of the Tac-ABCD chimera was notably more intense than that obtained with Tac-AB. Thus, the presence of a tetrameric coiled-coil following the CaM binding site stabilizes the AB/CaM complex.

The interaction of purified, monodispersed soluble GST-AB and GST-ABCD with D-CaM has been evaluated previously^32,41^. In contrast to GST-ABΔ2, D-CaM fluorescence emission increased to similar levels when probed with GST-AB or GST-ABCD (Supplemental Fig. 5), with a slightly higher apparent affinity for ABCD than AB (EC_50_ = 9.2 ± 0.1 and 11.0 ± 0.5 nM without Ca^2+^, p < 0.001; 15.1 ± 0.6 and 27.1 ± 1.2 nM in the presence of Ca^2+^, p < 0.001, respectively: see supplemental Table 1). Although an increase in apparent affinity is consistent with a stabilization of CaM binding by the coiled-coil domain, its small magnitude seemed insufficient to account for the dramatic increase in the signal seen in the pull-down experiments. Nevertheless, the combined data indicated that the presence of a tetramerization signal distal to the AB module significantly affects CaM binding.

**Figure 2:**
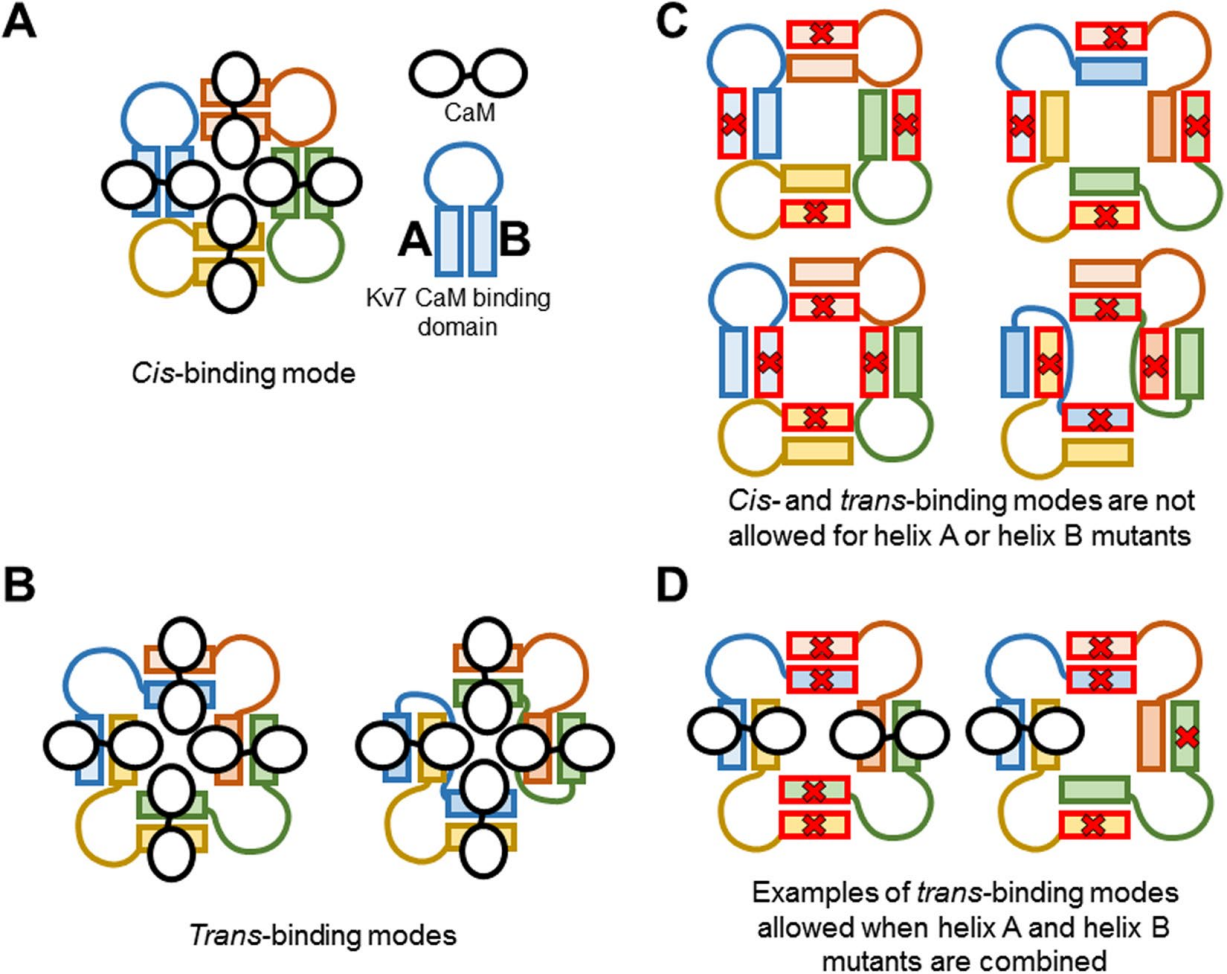
Hypothetical *cis* and *trans* calmodulin binding in tetramers. (**A**) A representation of *cis*-binding, in which CaM embraces helices A and B (*boxes*) in the same subunit. The N-lobe engages helix B, whereas the C-lobe engages helix A^37,42,43^. (**B**) Illustration of CaM in *trans*-binding mode embracing helices A and B from two different subunits (note that CaM embraces helices of different color). (**C**) Mutations in either helix A (top) or helix B (bottom) preclude *cis*- (left) and *trans*- (right) binding in homomeric tetramers. (**D**) *Trans*-binding is allowed between heteromeric helix A and helix B mutants, whereas *cis*-binding is precluded.

CaM could embrace helices A and B from the same subunit (*cis*-binding) or from two adjacent subunits (*trans*-binding: Fig. 2)^41,42^. Our working hypothesis is that by bringing together AB modules from adjacent subunits, the distal assembly domain favors *trans*-binding. Indeed, the atomic structure of the Kv7.1/CaM complex has been trapped bridging two CaM-binding domains^42^, although results obtained using concatenated constructs suggest that CaM preferentially binds to Kv7.1 channels in a *cis* configuration^42^. By contrast, there are functional indications for the adoption of *trans*-binding in Kv7.2 channels. Kv7.2 channels that carry a mutation in helix A or helix B, each of which disrupts CaM binding individually, are non-functional. Remarkably, when subunits carrying a mutation in helix A are co-expressed with subunits mutated in helix B, the resulting channel is functional, highlighting the relevance of CaM *trans*-binding^41^.

The *trans*-binding signal could be isolated by mixing an AB module carrying a mutation that precludes binding to helix A (A343D) with an AB module with a mutation that impedes binding to helix B (S511D)^41^. Neither of these mutants significantly increase D-CaM fluorescence in binding assays. By contrast, there was an increase in fluorescence when an equimolar mixture of both mutants was tested, indicating that CaM bridges the two proteins^41^ (see supplemental Fig. 6D).

Combining these mutant Kv7.2 CaM binding domains, we devised an assay to follow the adoption of the *trans*-binding mode *in vitro*, which involves monitoring D-CaM fluorescence while sequentially adding the mutant proteins. For simplicity, an equimolar mixture of the AB-A343D (helix A mutant) and the AB-S511D (helix B mutant) is denoted AB#, while ABCD# stands for an equimolar mixture of ABCD-A343D and ABCD-S511D. Please note that the concentrations refer to each mutated protein and thus, 100 nM of AB# is the result of mixing 100 nM of AB-A343D and 100 nM of AB-S511D. Consequently, the number of WT helices A and B should remain the same at a given concentration of AB and AB#, and consequently, the number of CaM binding sites is expected to be the same.

**Figure 3:**
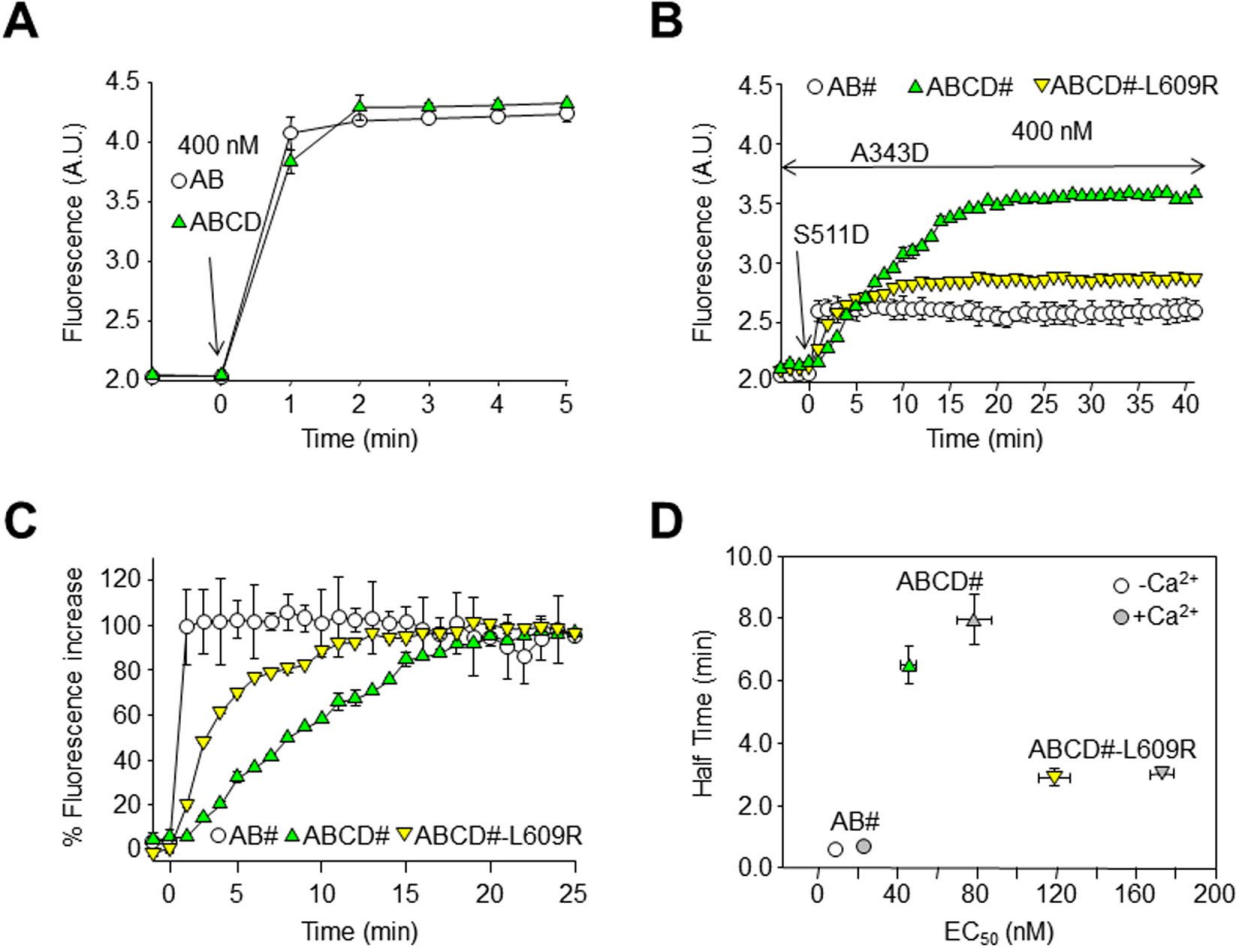
The time-course of *trans*-binding is affected by the helix D coiled-coil. (**A**) Time-course of the increase in D-CaM (12.5 nM) fluorescence upon binding to AB and to ABCD (400 nM). Each point represents the average of 4 experiments. The maximal increase in fluorescence was reached in less than 2 min. (**B**) Comparison of the time-course of the increase in D-CaM fluorescence in the continued presence of the helix A A343D mutant (400 nM), and upon addition of the S511D helix B mutant (400 nM), for proteins devoid of the CD module (AB#, open circles), ABCD# (green upward triangles), and ABCD#-L609R (yellow downward triangles). The hash denotes an equal mixture of helix A and helix B mutants. This set-up was designed to trap *trans*-binding. Each trace represents the average of 3 experiments. (**C**) Normalized time-course from the data displayed in (**B**). (**D**) Plot of the half-time to reach the maximal D-CaM fluorescence emission *vs* the apparent affinity for AB#, ABCD# and ABCD#-L609R.

We first evaluated binding to proteins devoid of mutations. Figure 3A shows the time-course of the increase in D-CaM fluorescence after adding the CaM binding AB module, as well as the time-course after adding the ABCD module that included the CD assembly region. After less than 2 min the signal approached the full response in both cases (see supplemental Fig. 7).

There were no significant changes in D-CaM fluorescence emission upon addition of the helix B S511D mutant (not shown, see^41,44^). Similarly, there were no significant changes in D-CaM fluorescence emission upon addition of the helix A A343D mutant (Fig. 3B). Remarkably, the subsequent addition of the helix B S511D mutant resulted in an increase in fluorescence within 1 min, a time-course that was beyond the resolution of our experimental set up. Thus, the acquisition of the *trans* configuration in solution (see supplemental Fig. 6) took less than 1 min in the absence of the assembly domain (Fig. 3B,C, *circles*).

We next tested the influence of helices CD on the time-course of the acquisition of *trans*-binding. Figure 3B,C (upward triangles) show that the presence of helices CD produced several changes in the profile of the response. First, the magnitude of the increase in fluorescence was larger and second, the time-course for *trans*-binding was slower. The extent of the fluorescent increase was indistinguishable at 25 °C and 37 °C, whereas the time-course was faster at 37 °C (Supplemental Fig. 8). The time to reach the half-maximal signal was 9.2 ± 0.3 and 12.0 ± 0.4 min, at 37 °C and 25 °C, respectively when the concentration tested was 400 nM.

The apparent binding affinity of an equimolar mixture of the helix A and helix B mutant proteins that are devoid of helices CD (AB#) was similar to that observed for the AB module (EC_50_ = 8.5 ± 1.1 nM [n = 4] for AB# [AB-A343D/AB-S511D] [Fig. 3D, circles], compared to 11.0 ± 0.5 nM for AB*;* see also supplemental Table 1). By contrast, the apparent binding affinity significantly decreased when helices CD were present (Fig. 3D, green upward triangles, EC_50_ = 45.5 ± 3.8 nM [n = 3] for ABCD# [ABCD-A343D/ABCD-S511D], compared to 9.1 ± 0.1 nM for ABCD*;* supplemental Table 1). To further test the role of tetrameric coiled-coil formation by helix D, the impact of introducing the coiled-coil disrupting L609R mutation was studied^32^ (Supplemental Fig. 8). Two major differences became apparent as the time to reach maximal fluorescence and the magnitude of the maximal change were reduced compared to that of WT ABCD (Fig. 3B,C, yellow downward triangles). In addition, the apparent binding affinity was lower (EC_50_ = 119.5 ± 8.1 nM, [n = 3]) compared to ABCD# (EC_50_ = 45.5 ± 3.8 nM, [n = 3]; Fig. 3D, downward vs upward triangles; see also supplemental Table 1). Figure 3C shows the normalized time-course of *trans*-binding, illustrating that the rate was influenced by the assembly domain, and Fig. 3D shows the lack of correlation between the apparent binding affinity and the rate at which fluorescence increased. Thus, the changes in apparent affinity failed to explain the differences in kinetics.

**Figure 4:**
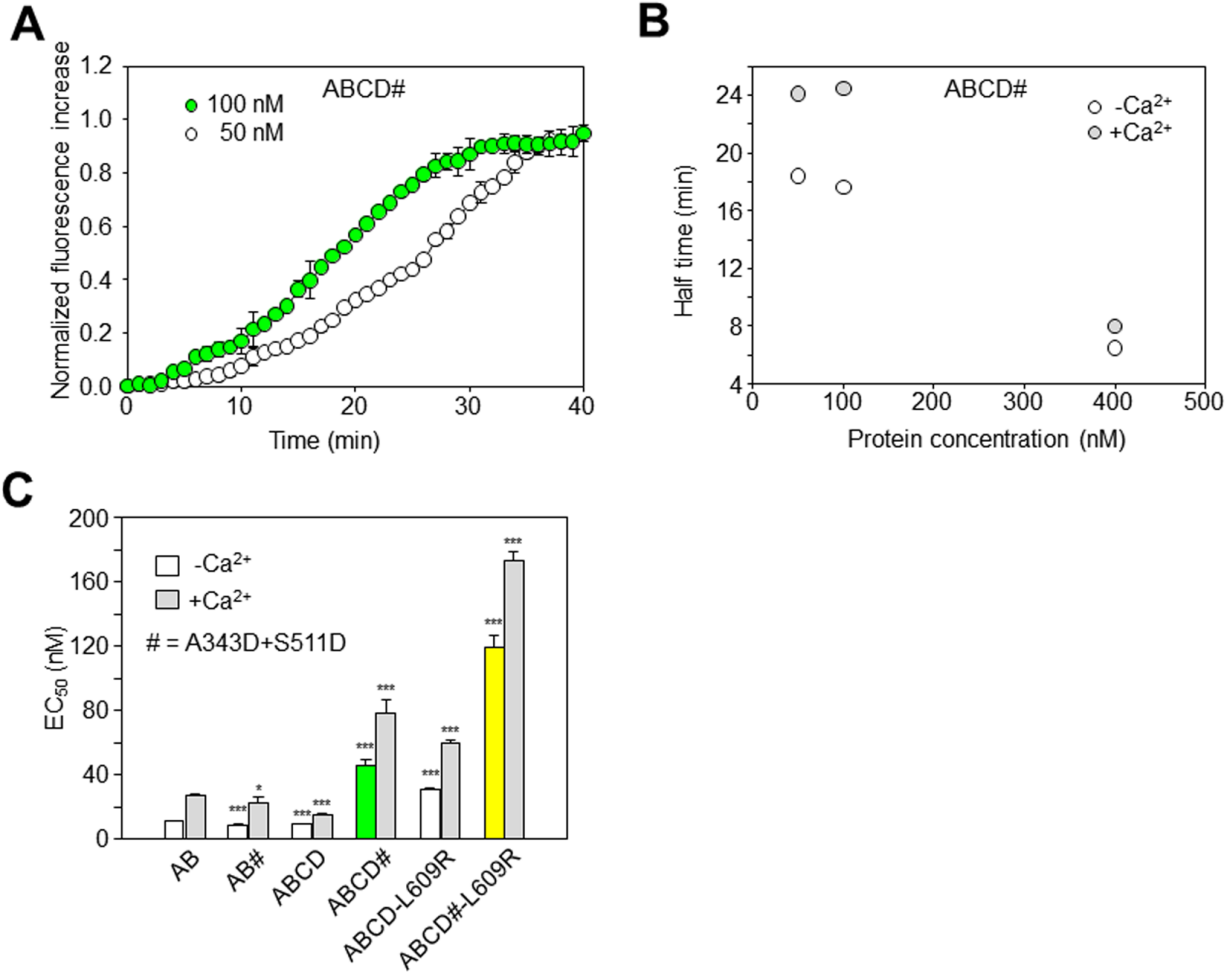
Acquisition of the *trans*-binding mode depends on the protein concentration and calcium levels. (**A**) Time course of the increase in D-CaM fluorescence obtained upon binding to ABCD# (50 or 100 nM). Each trace represents the average of 3 experiments. (**B**) Relationship between the time to reach the half-maximal increase in D-CaM fluorescence and protein concentration in the presence (gray circles) and absence of Ca^2+^ (white circles). (**C**) Plot of the apparent binding affinity (EC_50_) obtained from the concentration-response curves as in Fig. 1B (D-CaM 12.5 nM). The apparent binding affinity was derived from 3 or more experiments. The experiments were performed both in the presence (gray bars) and absence of Ca^2+^ (white, yellow and green columns). The asterisks indicate significantly different values versus AB: *p < 0.05; ***p < 0.001.

As expected, the rate of adopting the *trans*-binding mode was dependent on the concentration of the ABCD# proteins (Fig. 4A,B). The latency in the time-course was more apparent at lower protein concentrations, indicating that there are multiple binding steps before D-CaM adopts a conformation that enhances fluorescence emission. The impact of Ca^2+^ was evaluated in the complementation assay with mutant ABCD proteins, indicating that Ca^2+^ significantly reduced the rate of the increase in D-CaM fluorescence. The Ca^2+^-dependency of the rate of *trans* binding was evident at every protein concentration tested (Fig. 4B). In addition, the apparent binding affinity was lower in the presence of Ca^2+^ for all the constructs tested (Fig. 4C), in line with previous observations^45,46^.

**Figure 5:**
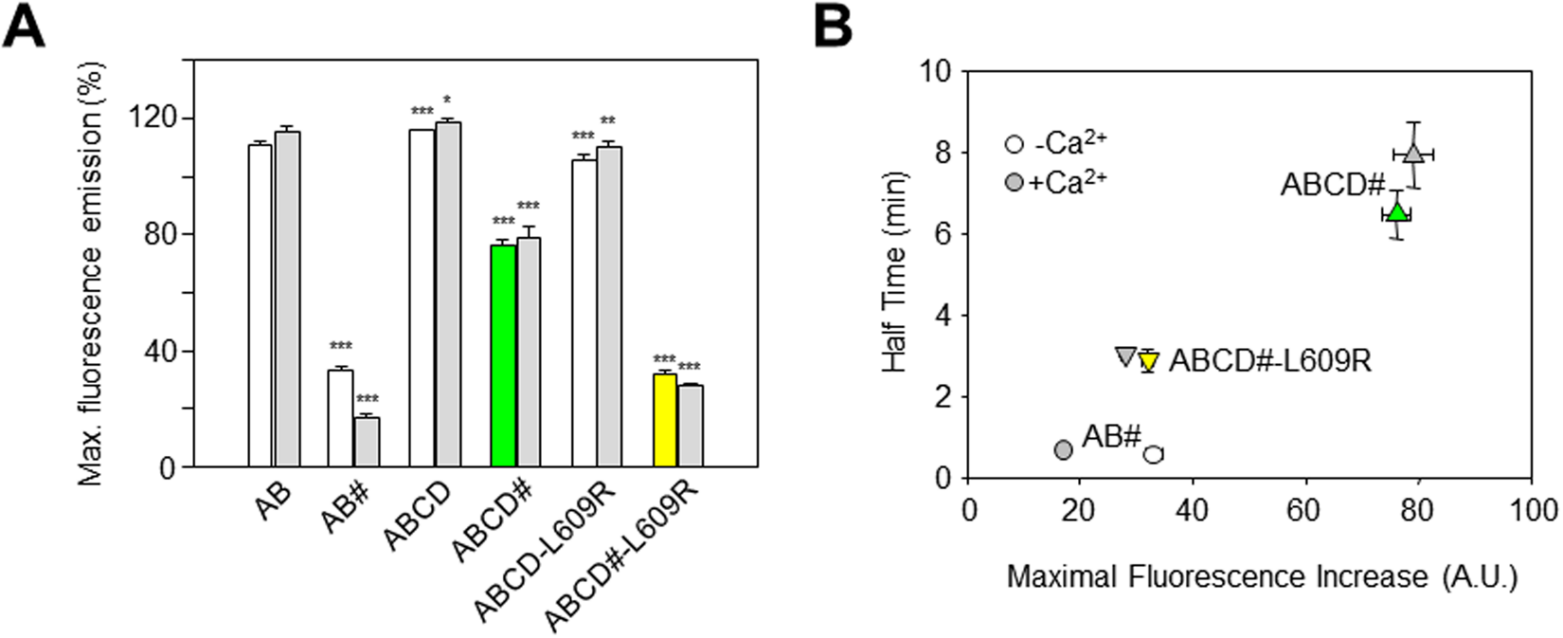
Summary of the maximal D-CaM fluorescence emission. (**A**) Maximal increases in D-CaM fluorescence emission induced by saturating concentrations of the indicated proteins. The data were collected in the presence (gray bars) or absence of Ca^2+^ (white, yellow and green bars) (n ≥ 3). Asterisks indicate significantly different values versus AB: *p < 0.05; **p < 0.01; ***p < 0.001. (**B**) Plot of the time to reach the half-maximal D-CaM fluorescence versus the maximal increase in fluorescence.

Finally, the maximal increase in fluorescence was reduced in the complementation assay (AB *vs* AB#, ABCD *vs* ABCD#, ABCD-L609R *vs* ABCD#-L609R; Fig. 5A), which was partially related to the kinetics of the increase in D-CaM fluorescence (Fig. 5B). Precluding *cis*-binding mode caused a reduction of about 25% in the maximal D-CaM fluorescence, as revealed by comparing ABCD and ABCD# (Fig. 5A). Except for AB#, the magnitude of this reduction was not affected by Ca^2+^ and it was larger ( > 65% reduction) when the tetramerization domain was absent (AB *vs* AB#) or when there was a mutation that was expected to preclude helix D coiled-coil formation (ABCD *vs* ABCD#-L609R).

When comparing data obtained with ABCD and ABCD#, it should be considered that ABCD# could adopt either a configuration displaying the same number of CaM binding sites as ABCD (Fig. 2D, left) or only half the binding sites (Fig. 2D, right). If both binding modes were present in equal proportions, the maximal fluorescence for ABCD# should be about 75%, not far from the value actually observed (Fig. 5A). However, additional factors are required to explain the weaker fluorescence observed for AB# and ABCD#-L609R. We previously proposed that the interaction between AB and D-CaM is best described as a two-step process, in which D-CaM first binds to AB to produce AB-CaM, and in the second step, D-CaM undergoes a conformational change that leads to increased fluorescence, a state defined as AB-CaM*^36^. In the context of this model, the simplest explanation is that the absence of helix D or its disruption by the L609R mutation reduced the stability of AB-CaM* in the *trans*-binding mode.

The relationship of half-time to the maximal increase in D-CaM fluorescence shown in Fig. 5B reveals that the slower the process, the larger the increase in fluorescence. This result is what would be expected if an increase in the stability of the tetrameric structure led to a lower rate of CaM exiting the complex. Accordingly, the variations in maximal fluorescence are consistent with the idea that helix D coiled-coil formation helps stabilize the AB/CaM complex.

**Figure 6:**
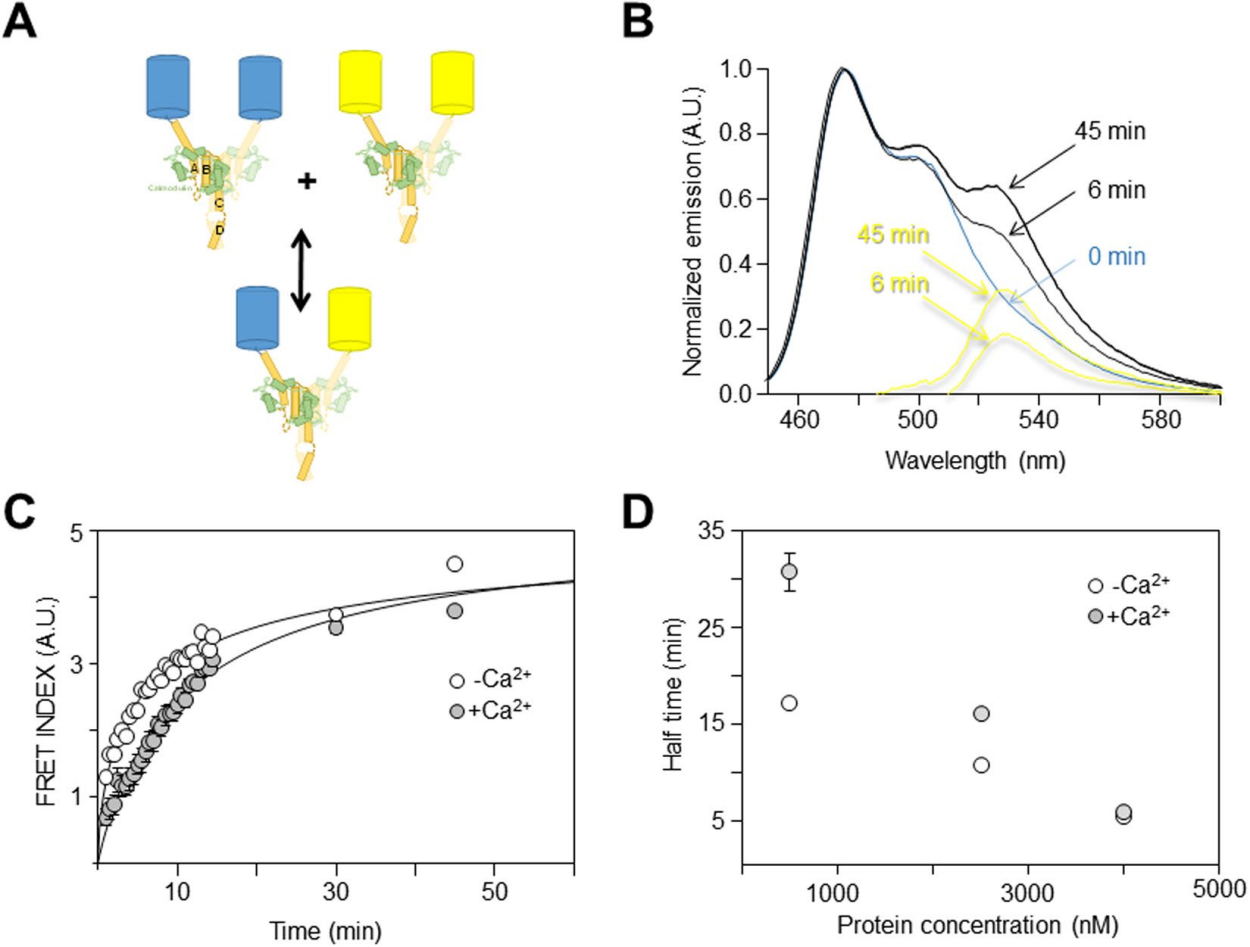
The time-course of subunit exchange between FP-ABCD/CaM tetrameric complexes is affected by calcium. Two ABCD/CaM complexes with a fluorescent protein attached to the N-terminus (CFP, donor; YFP, acceptor) were purified and the development of FRET was monitored over time from equimolar mixtures. (**A**) Cartoon representing the experiment: the CFP-ABCD/CaM complex was mixed with YFP-ABCD/CaM, resulting in an exchange of proteins that led to the development of FRET. Only two subunits of the tetrameric complexes are drawn for clarity. (**B**) Normalized emission spectra of a mixture of 2.5 µM CFP-ABCD/CaM and 2.5 µM YFP-ABCD/CaM at different times. The yellow traces are the results of subtracting the normalized CFP emission spectra and isolating the emission of the acceptor (YFP). (**C**) Time course of the increase in the FRET index from a 2.5 µM CFP-ABCD/CaM and 2.5 µM YFP-ABCD/CaM mixture in the presence (gray circles) and absence (white circles) of Ca^2+^. Each trace represents the average of 3 experiments. (**D**) Relationship between the time to reach the half-maximal increase in the FRET index and the protein concentration in the presence (gray circles) and absence of Ca^2+^ (white circles). Each point represents the average of 3 or more experiments.

To gain further insight into the relationship between CaM binding and tetramerization of the wt ABCD domain, the interchange between tetrameric assemblies was monitored by FRET. In the presence of CaM, we produced and purified two versions of the ABCD domain tagged at the N-terminus with cyan (the CFP turquoise2 variant) or yellow (the YFP citrine variant) fluorescent proteins (Fig. 6; Supplemental Fig. 9). These conditions do not distinguish the CaM binding mode and indeed, they could support both *cis-* and *trans-*CaM binding. It was previously shown that these proteins behave as tetramers with 4:4 ABCD/CaM stoichiometry^5,32^. After mixing both proteins, there was a time dependent increase in the 530 nm emission of the acceptor upon excitation at 436 nm, suggesting that exchange between subunits of tetrameric complexes was taking place. The development of FRET over time in an equimolar mixture of the CFP-ABCD/CaM complex (donor) and the YFP-ABCD/CaM complex (acceptor) is shown in Fig. 6. The time to reach the half maximal FRET index was 12.2 ± 1.6 min for a 500 nM mixture. Assuming that the exchange between the ABCD domains is the rate-limiting step for both the complementation and FRET assays, comparing between the rates obtained under both paradigms would seem to be feasible. The time to reach the half maximal FRET at 500 nM was about twice that for ABCD# proteins at 400 nM. When the donor and acceptor concentration was raised 5-fold (2.5 µM), the time to reach half maximal FRET was reduced to 6.9 ± 0.2 min, comparable to the 6.5 ± 0.1 min half-time observed in the *trans*-binding assay at 400 nM (Fig. 4B, i.e. a six-fold difference in protein concentration). Thus, the introduction of mutations into the CaM binding site led to a faster exchange (in the complementation assay), meaning that those mutations perturbed the stability of the tetrameric assembly.

The time-course of FRET development was sensitive to Ca^2+^, taking longer to reach the half-maximal value in the presence of this cation (Fig. 6C,D). This is similar to the response in conditions designed to monitor *trans*-binding (Fig. 4B), indicating that the ABCD/CaM complexes were more stable in the presence of Ca^2+^. Thus, the stability of the C-terminal tetrameric complex is sensitive to Ca^2+^ oscillations in the physiological range, probably through the binding of this cation to CaM. This is particularly remarkable, considering that helix D and the AB CaM-binding domain lie more than 20 Å apart according to cryo-EM images, with no evidence of direct physical interactions between helix D and either CaM or the AB domain^47^ (see supplemental Fig. 1).

Our data shows that exchange between ABCD domains does occur in solution, yet how the transmembrane region of the channel, absent in the isolated domains, influences the exchange and flexibility of the assembly domain remains unclear. Recent cryo-EM images of the Kv7.1/CaM complex in membranes show an interaction between the third EF hand of CaM and the S2-S3 loop of Kv7.1^47^. How this interaction affects the propensity to adopt the *trans-* or *cis*-CaM binding is still not known. In addition, and possibly because of this interaction, Ca^2+^ does not occupy the third EF hand. Thus, it might be expected that the dependence on Ca^2+^ to adopt *trans* and *cis* binding might differ substantially in the complete channel relative to the GST-tagged-Kv7.2 C-terminal constructs studied here. Interestingly, these cryo-EM images reveal that the helix D region is very flexible^47^, an observation in line with the exchange between subunits observed here for the isolated C-terminal domain. Is the exchange of helix D between adjacent channels feasible? For that to take place, channels must be in close proximity. Often channels are organized in clusters^48–50^, and Kv7.2 are clustered in the axonal initial segment and nodes of Ranvier^51–53^, raising the possibility that Kv7.2 channels do come into close proximity. If two channels are adjacent to each other, the distance between their helix D domains will be in the order of 70 Å^47^ (see supplemental Fig. 1), which is the average distance between two molecules at concentrations in the millimolar range. Based on the kinetic information we obtained, if channels are clustered they can communicate through helix D in a sub-second timescale. However, further experiments will be necessary to address the occurrence and implications of such hypothetical exchanges between neighboring channels.

Our studies suggest a mechanism by which cytoplasmic factors can modulate channel gating by altering the stability of the helix D coiled-coil, thereby influencing CaM-dependent PIP_2_ regulation^32^. The discovery that the helix D coiled-coil domain can affect Kv7.2 channel gating suggests that a similar mechanism regulating the gating of channels with equivalent CaM binding and coiled-coil domain architecture is feasible^4,16,17,20^. Thus, it would be of interest to explore the coupling between assembly and the CaM-dependent regulation of gating of these different ion channels.

In summary, the data presented here reveal that helix D-dependent coiled-coil formation stabilizes the interaction between CaM and helices A and B. Thus, it is possible that CaM binding mediates some of the effects in channel processing, surface expression and gating that originate from alterations to helix D. This stabilization may arise in part from the geometric configuration, by helix D promoting the formation of a more compact AB/CaM ring under the pore, which in turn could allow CaM to engage helices A and B in different modes of binding. Concurrently, CaM influences the stability of the tetrameric helix D coiled-coil, conferring Ca^2+^-dependency to the assembly of the C-terminal domain. Our observations are consistent with a model that involves active reciprocal coupling between the A-B module and the tetramer formed by helices D, CaM and Ca^2+^ thereby influencing the dynamic behavior of the helix D coiled-coil.

## Materials and Methods

### Molecular biology

The human Kv7.2 (Y15065) cDNA was provided by T. Jentsch (Leibniz-Institut für Molekulare Pharmakologie, Berlin, Germany) and the cDNA encoding rat CaM was provided by the group of J.P. Adelman (Vollum Institute, Portland, OR, USA). Deletions, point mutations and epitope insertions were generated by PCR-based mutagenesis.

### Cell culture and transfection

HEK293T cells (HEK 293 T/17, ATCC, CRL-11268) were maintained at 37 °C/5% CO_2_ in Dulbecco’s Modified Eagle’s Medium (DMEM, Sigma-Aldrich), supplemented with non-essential amino acids (Sigma, Madrid, Spain) and 10% FBS (Lonza, Madrid, Spain). Cells were transiently transfected with cDNAs using calcium phosphate or PEI 25000 (PolySciences ref: 23966-2).

### Antibodies

The primary monoclonal antibodies used here were a mouse anti-CaM (1:2000; 05-173, Millipore) and a mouse monoclonal anti-GFP (1:2000; clones 7.1 and 7.3, 11814460001, Roche Applied Science, Penzberg, Germany). The peroxidase-coupled secondary antibody used was an anti-mouse IgG (1:5000; Bio-Rad Laboratories, Hercules, CA, USA). Proteins were visualized using the SuperSignal West Pico Chemiluminescent Substrate (34078, Pierce). At least 10 cumulative images (30 s exposition) were acquired using the Versadoc Imaging System (Bio-Rad Laboratories, Madrid, Spain) and the protein bands were analyzed using ImageJ software v1.45.

### Immunoprecipitation

For immunoprecipitation experiments we used the Tac-CFP construct generated using the monomeric Tac receptor described previously^33^. Different fragments of Kv7.2 were inserted between Tac and mCFP. Where indicated, a deletion between helices A and B was introduced (Δ2: Del T359-T501)^34^ and helix D was replaced by an artificial tetramerization (Tet) signal^35^ where indicated. Thirty-six hours after transfection, HEK293T cells were solubilized for 30 min at 4 °C in IP buffer containing Tris-HCl 50 mM, NaCl 150 mM, Triton X-100 1%, EDTA 2 mM, EGTA 5 mM and protease inhibitors (1X Complete; Roche). The nuclei were pelleted at 500 g for 3 min and the supernatant was then centrifuged at 11,000 g for 20 min to remove the insoluble material. Lysates were precleared with 40 ml of equilibrated Protein A sepharose beads (Sigma P3391) for 1 h at 4 °C. Anti-GFP antibodies were immobilized on 40 ml of equilibrated Protein A beads overnight at 4 °C and washed twice with IP buffer. Precleared lysates were incubated overnight at 4 °C with Protein A-anti-GFP and after 4 washes with IP buffer, the immunoprecipitated proteins were recovered by heating at 90 °C for 5 min in sodium dodecyl sulfate (SDS) sample buffer.

### Recombinant protein production

Protein expression and purification protocols for Kv7.2 helices AB and the helices ABCD fused to GST (GST-AB and GST-ABCD), the deletions and mutants (as indicated in the figures), and for CaM have been described in detail elsewhere^36,37^. Proteins were checked for purity by Coomassie brilliant blue staining of 10 or 15% SDS-PAGE gels. In order to exclude the presence of aggregates, the oligomerization state of the purified proteins was examined by dynamic light scattering (DLS) using a Zetasizer Nano instrument (Malvern Instruments Ltd.). Samples were filtered through 0.22 μm membrane filters (Millipore) and centrifuged at 13,000 g for 10 min. Samples placed onto single use plastic cuvettes were maintained at a fixed temperature of 25 °C. The protein concentration was 1 mg/ml and the buffer used was Tris-HCl 20 mM, NaCl 100 mM [pH 7.5]. Measurements were made at an angle θ = 90° to the incident beam and the data were collected every 60 s. The correlation functions were analyzed to obtain the distributions of the decay rates and hence, the apparent diffusion coefficients, and ultimately the distributions of the hydrodynamic radius of the scattering particles in solution were obtained via a Stokes-Einstein equation. Finally, the monodispersity or polydispersity of the solutions was assessed and the molecular weights of the predominant species were calculated.

### Fluorometric measurements using dansyl-CaM

Fluorescent dansylated CaM (D-CaM, 5-(dimethylamino)naphtalene-1-sulfonyl-calmodulin) was prepared using recombinant CaM and dansyl chloride, as described previously^38,40^. Prior to the experiments, D-CaM and other proteins were dialyzed for 48 h against 2 L of the Fluorescence buffer containing Tris-HCl 25 mM [pH 7.4], KCl 120 mM, NaCl 5 mM, MgCl_2_ 2 mM_,_ EGTA 10 mM, changing the buffer every 12 h. Steady-state fluorescence measurements were obtained at 25 °C on an Aminco Bowman series 2 (SLM Aminco) fluorescence spectrophotometer in a final volume of 100 μl (using quartz cuvettes). Time course experiments were performed at 25 °C and 37 °C, with excitation at 340 nm and emissions recorded from 400 to 660 nm (titration experiments) or at 500 nm (time trace). Slit widths were set at 4 nm for excitation and 4 nm for emission.

Titration experiments were performed by adding increasing concentrations of each fusion protein to a cuvette containing D-CaM (12.5 nM) in Fluorescence buffer. Experiments were also performed in the presence of an excess of free Ca^2+^ (3.9 µM) by adding 9.63 mM Ca^2+^ to the Fluorescence buffer. The free Ca^2+^ concentration was determined using Fura-2 (Invitrogen), following the manufacturer’s instructions.

For the time course experiments, the dansyl emission of D-CaM (at 500 nm) was measured as a function of time (min). In these experiments, a mutated protein (400 nM) was added to a stirred cuvette containing D-CaM (12.5 nM) in Fluorescence buffer (in the presence or absence of Ca^2+^) and after two minutes, another mutant (400 nM) was added. Finally, the experiments involving GST-AB wt and GST-ABCD wt were conducted by adding 400 nM of these proteins to D-CaM (12.5 nM).

Fluorescence enhancement was plotted against the protein concentration to generate the concentration-response curves, or in the case of the time course experiments, it was plotted against time (min) to obtain the time-response curves. The parameters of the Hill equation were fitted to the data by curvilinear regression, enabling the apparent affinity (EC_50_ or concentration that gives half-maximal change in the intensity of the fluorescence emission) or the t_50_ (half-time or time that gives half-maximal change in the intensity of fluorescence emission). The data are shown as the average of 3 or more independent experiments.

### FRET

The fluorescent mTurquoise2 (donor) and mCitrine (acceptor) proteins were fused to the N-terminal of Kv7.2-ABCD (residues 310-653), and cloned into a pProEX-HTc plasmid (Invitrogen) that introduces a 6xHis N-terminal tag. CaM was cloned into the co-expression compatible plasmid pOKD4^54^ and both plasmids were co-transformed by electroporation in BL21(DE3) cells (Novagen). Cells were grown at 37 °C in 1 L of LB medium containing ampicillin and kanamycin until an A_600_ = 0.6–0.8 was reached. The expression of the fusion proteins was induced with 0.3 mM of IPTG O/N at 20 °C. The cells were then harvested by centrifugation at 9,000 g for 9 min and re-suspended in 25 ml of Buffer A (KCl 120 mM, K-HEPES 50 mM [pH 7.4], imidazole 20 mM, DTT 500 μM, PMSF 1 mM, protease inhibitor EDTA free: Roche, Ref. 04693132001). After lysis by sonication (15 s ON, 15 s OFF, 10 cycles), the slurry was centrifuged at 25,000 g for 30 min, and the supernatant was filtered (0.20 μm) and transferred to a clean tube. The complex was affinity purified from the supernatant using a His-Trap-talon column and equilibrated with FRET buffer (KCl 120 mM, Hepes 50 mM, NaCl 5 mM, EGTA 5 mM). The fractions containing soluble monomeric C-terminal proteins were identified by SDS-PAGE. Size-exclusion chromatography was performed using Superdex 200 pg 26/60 column (GE Healthcare, ref. 28–9893) pre-equilibrated with KCl 120 mM, HEPES 50 mM [pH 7.4], NaCl 5 mM and EGTA 5 mM. Fractions containing the protein complex were concentrated using Amicon Ultra-15 centrifugal filter units with a 3 kDa cut-off (Sigma-Aldrich).

All FRET experiments were carried out on an Aminco Bowman series 2 (SLM Aminco) luminescence fluorimeter, using quartz cuvettes (light width 3 mm, 0.1 mL volume). The Ca^2+^ concentration was calculated using Maxchelator Ca-EGTA calculator v1.3 (maxchelator.stanford.edu/CaEGTA-TS.htm). The samples were centrifuged at 14,000 g for 10 min to remove any aggregates formed. Each sample was excited at 433 nm (4 nm slit) and the emission spectra was collected from 450 to 600 nm (4 nm slit). The emission spectra was normalized to the peak emission at 476 nm. The normalized emission spectrum of turquoise2-ABCD was subtracted from each normalized spectra resulting in the isolation of the normalized emission spectra from the acceptor (see yellow traces in Fig. 6A). A FRET index was obtained as the integral from 524 to 538 nm of the normalized acceptor emission.

### Statistics

The data are expressed as the mean ± SEM and significant differences between the data (p < 0.05) were evaluated with the Student’s t-test: ***, significance at p < 0.001, **p < 0.01, and *p < 0.05.

### Availability statement

Materials, data and associated protocols will be made available on request.

## Electronic supplementary material

Supplemental Information
