# Supplementary material for: Calmodulin confers calcium sensitivity to the stability of the distal intracellular assembly domain of Kv7.2 channels
Source: Sci Rep. 2017 Oct 18;7:13425. doi: 10.1038/s41598-017-13811-4 (PMC5647379; doi:10.1038/s41598-017-13811-4)
Supplement: Supplementary file 1 — Supplemental Information [file 41598_2017_13811_MOESM1_ESM.pdf]

SUPPLEMENTARY DATA SET

Calmodulin confers calcium sensitivity to the stability of the distal intracellular assembly domain of Kv7.2 channels

Alessandro Alaimo, Eider Nuñez, Paloma Aivar, Juncal Fernández-Orth, Carolina Gomis-Perez, Ganeko Bernardo-Seisdedos, Covadonga Malo and Alvaro Villarroel‡

Instituto Biofisika, Consejo Superior de Investigaciones Científicas, CSIC, UPV/EHU, Barrio Sarriena s/n, 48940 Leioa, Spain.

Alessandro Alaimo	alessalaimo@gmail.com<br>execution and interpretation
Eider Nuñez	enviadero@gmail.com<br>execution
Paloma Aivar	aivar.mateo.p@gmail.com<br>execution
Juncal Fernandez-Orth	juncalforth@hotmail.com<br>execution
Carolina Gomis-Perez	karolgomis@gmail.com<br>reagents
Ganeko Bernardo-Seisdedos	ganeko7@gmail.com<br>reagents
Covadonga Malo	covadonga.m@gmail.com<br>reagents
Alvaro Villarroel	alvaro.villarroel@csic.es<br>conception, design, interpretation

Running title: Reciprocal calmodulin-tetramerization influences the C-terminal domain of Kv7.2 channels

‡To whom correspondence should be addressed: Alvaro Villarroel, Instituto Biofisika, Consejo Superior de Investigaciones Científicas, CSIC, UPV/EHU, Universidad del País Vasco, Barrio Sarriena s/n, 48940 Leioa, Spain. Tel +34 94 601 3225. FAX +34 94 601 3360. E-mail: alvaro.villarroel@csic.es

Keywords: calmodulin, coiled-coil, KCNQ, M-current

Conflict of Interest: The authors declare no competing financial interests

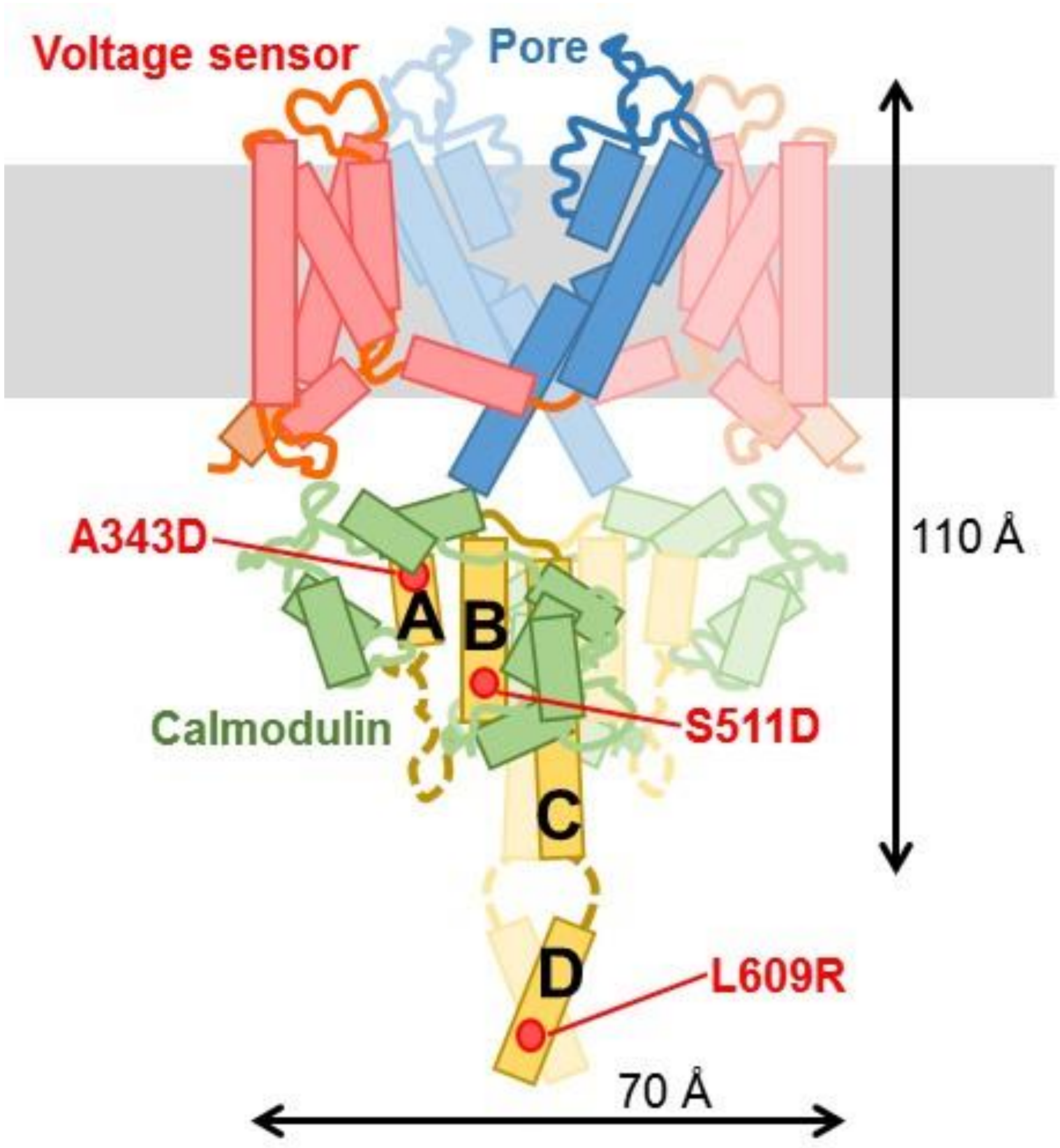

Suppl. Fig. 1. Cartoon representation of a KCNQ channel based on the flat projection of the atomic structure of Kv7.1 complexed with CaM[1]. The alpha helices are represented as rectangles and only two subunits of the tetramer are displayed. Some helices appear relatively shorter (e.g. Helix A) due to the projection of tilted helices on a surface. The approximate location of the helix A A343D and helix B S511D mutants that preclude CaM binding, and the helix D L609R that disrupts coiled-coil formation, are indicated. Because most of the linker between helices A and B is missing in the Kv7.1/CaM cryo-EM derived structure, this linker is only partially represented in this cartoon.

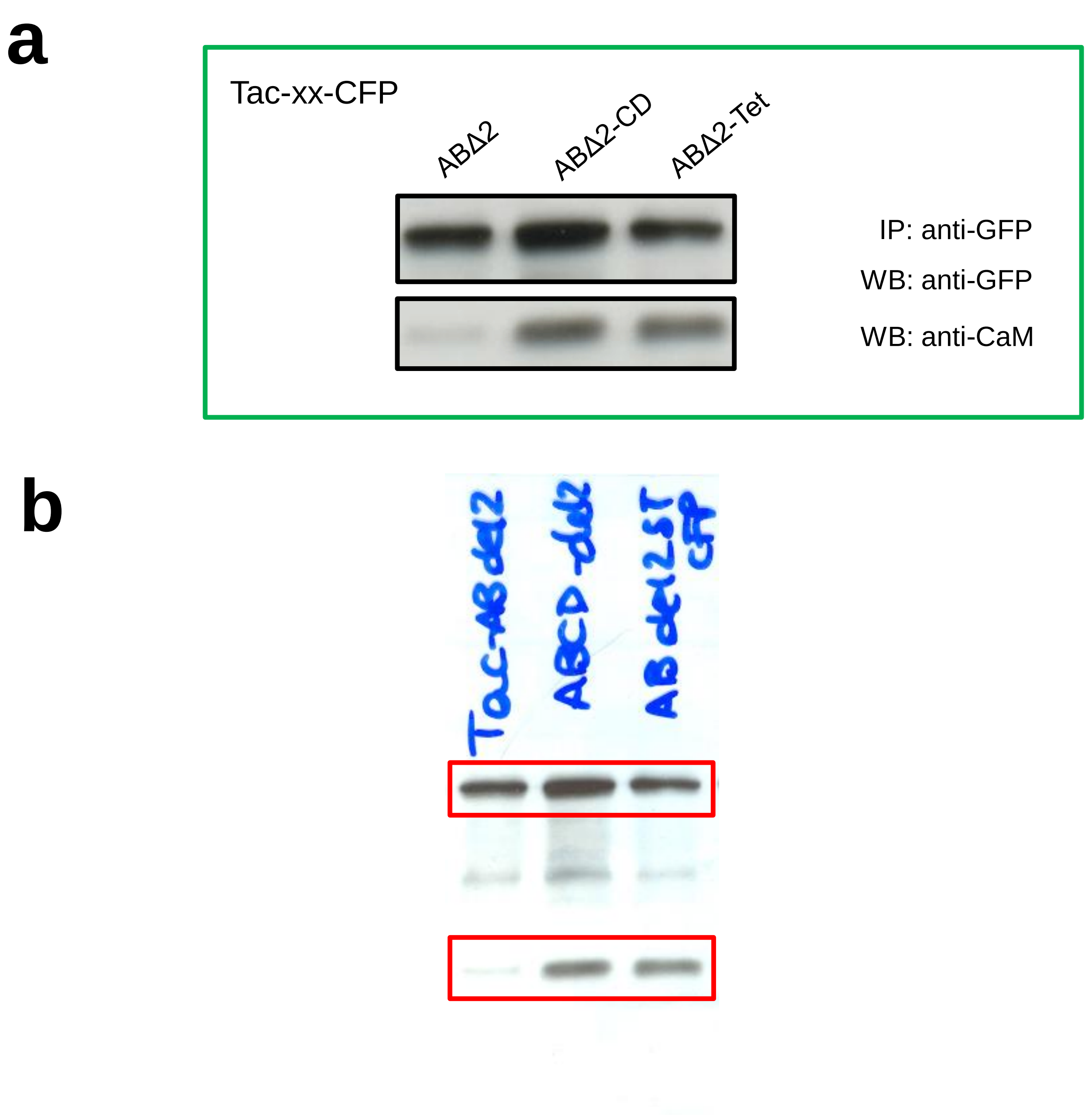

Suppl. Fig. 2. Full-length blots of the cropped images shown in Fig. 1A. (**a**) Fig. 1A of the manuscript contains images cropped from the full-length blots presented here (**b**).

a

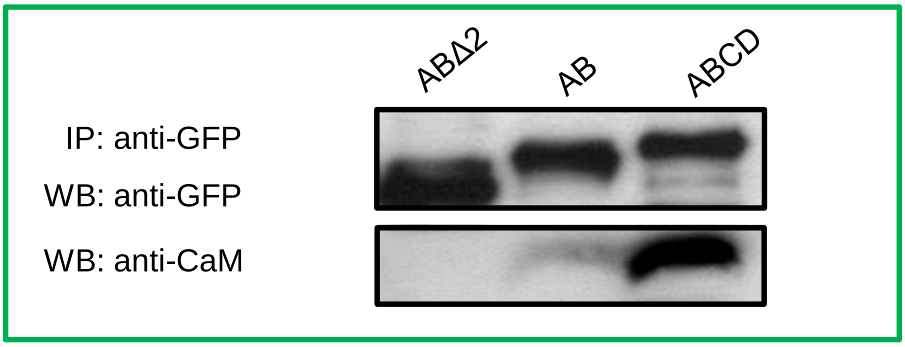

b

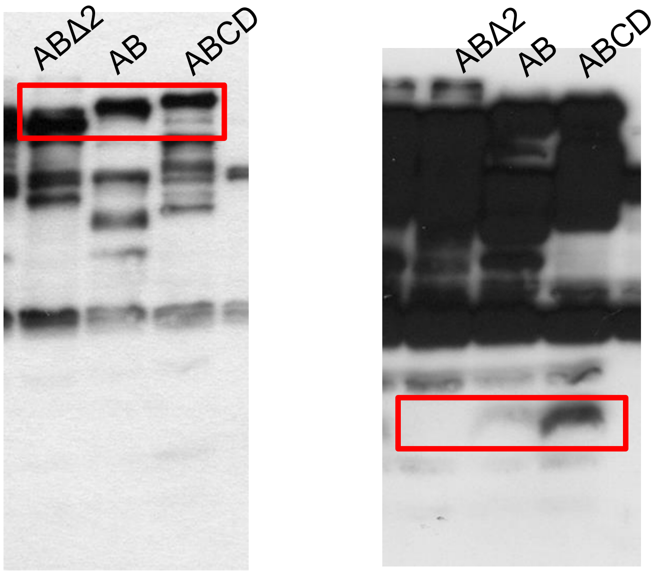

Suppl. Fig. 3. Full-length blots of the cropped images shown in Fig. 1C. (**a**) Fig. 1C of the manuscript contains images cropped from the full-length blots shown in **b** at two different exposures.

A

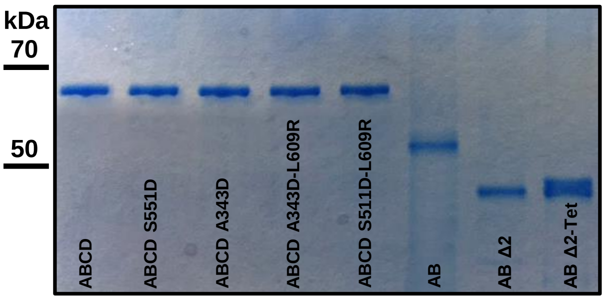

B

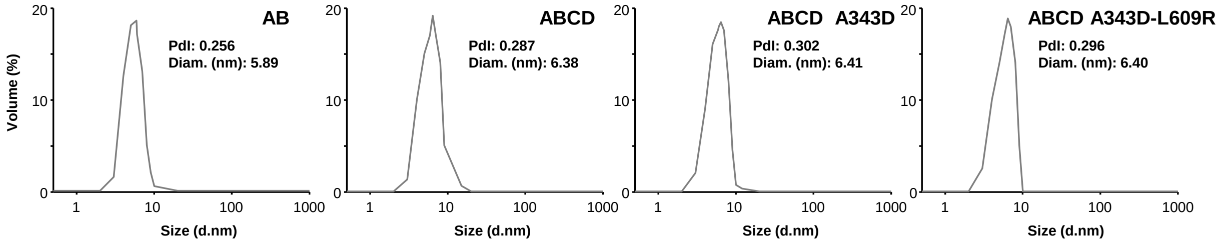

Suppl. Fig. 4. (**a**) SDS-PAGE analysis of the GST fusion proteins employed in this study (10% gel). (**b**) Representative dynamic light scattering (DLS) volume-weighted size distribution of 1 mg/ml of the indicated proteins. The buffer used contained Tris-HCl 20 mM and NaCl 100 mM [pH 7.5]. A low Polydispersity Index (PdI) was estimated from the apparent diameters assuming a globular protein shape and the unique peak argues against the presence of aggregates in these samples.

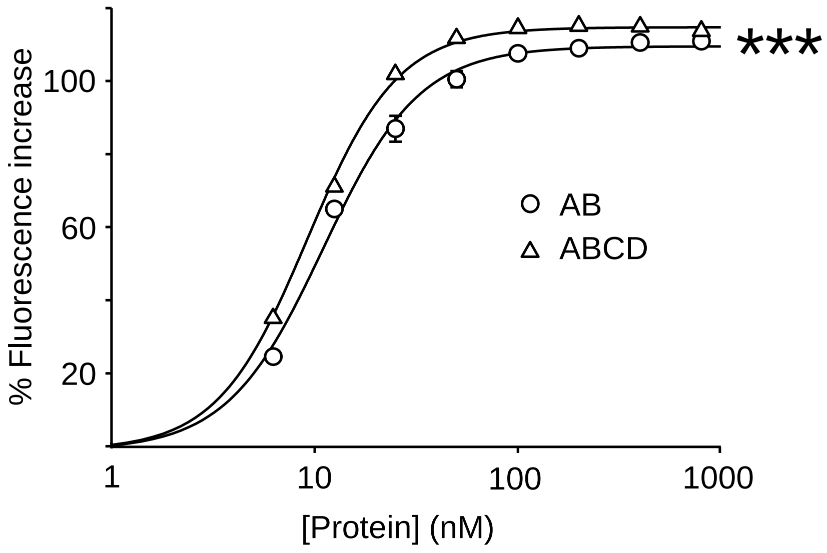

Suppl. Fig. 5. Concentration-dependent D-CaM (12.5 nM) fluorescence enhancement in the absence of Ca^{2+} (10 mM EGTA) for the indicated proteins. The lines are the result of fitting a Hill equation to the data. The EC_{50} (nM) was 11.0 ± 0.5 for AB and 9.1 ± 0.1 for ABCD. The asterisks indicate significantly different values of maximal fluorescence (*** = $p < 0.001$). The data represent the means ± SEM from 4 or more independent experiments: the data for AB is taken from[2] and that for ABCD from[3].

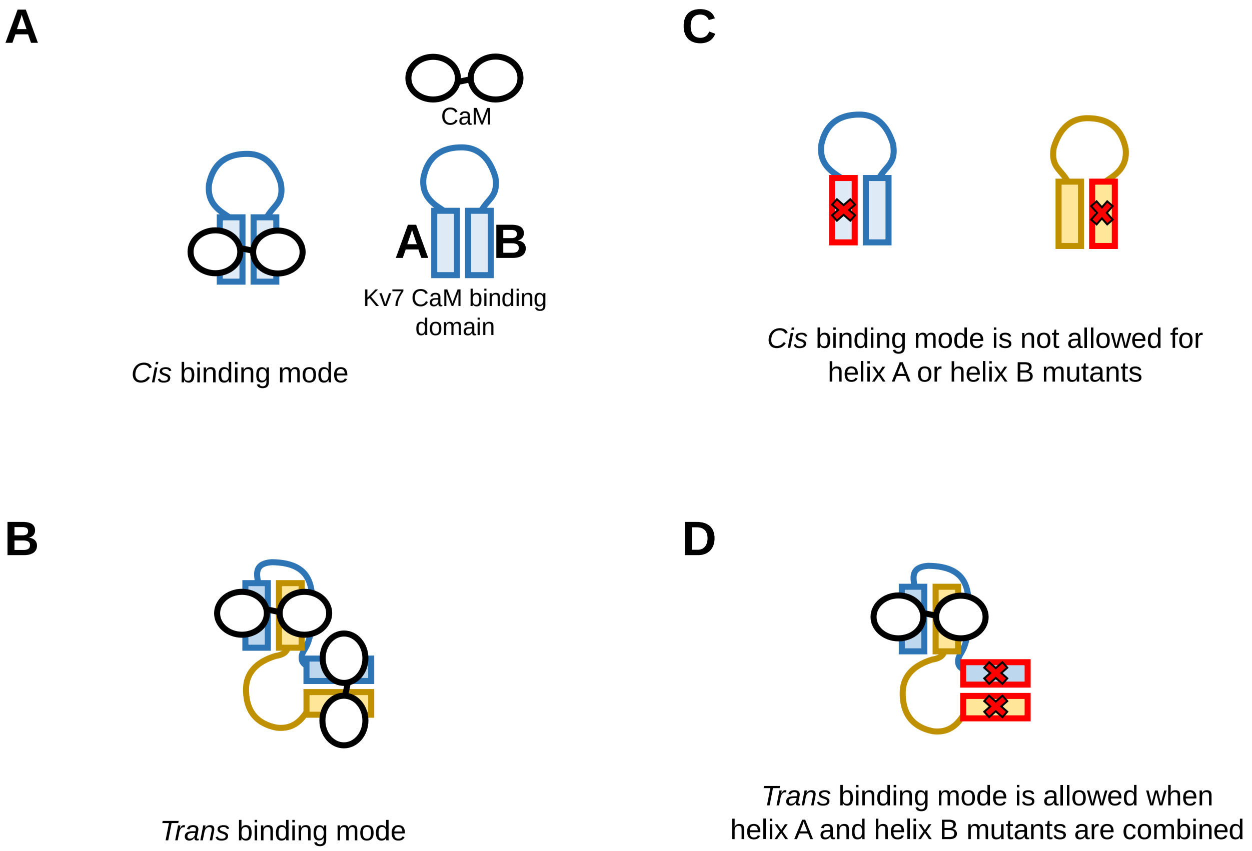

Suppl. Fig. 6. Hypothetical *cis*- and *trans*-calmodulin binding modes in the absence of helix D. (**a**) Representation of the *cis*-binding mode in which CaM embraces helices A and B (*boxes*) from the same subunit. The N-lobe engages helix B, whereas the C-lobe engages helix A[4-6]. (**b**) Illustration of CaM in the *trans*-binding mode, embracing helices A and B from two different subunits (note that CaM is embracing helices of different color). This arrangement has been observed in the crystallographic structure of the KCNQ1-AB/CaM complex[5]. (**c**) Mutations in either helix A (left) or helix B (right) preclude *cis*- (left) and *trans*- (right) binding. (**d**) In a mixture of helix A and helix B mutants *trans*-binding can occur, whereas *cis*-binding is precluded.

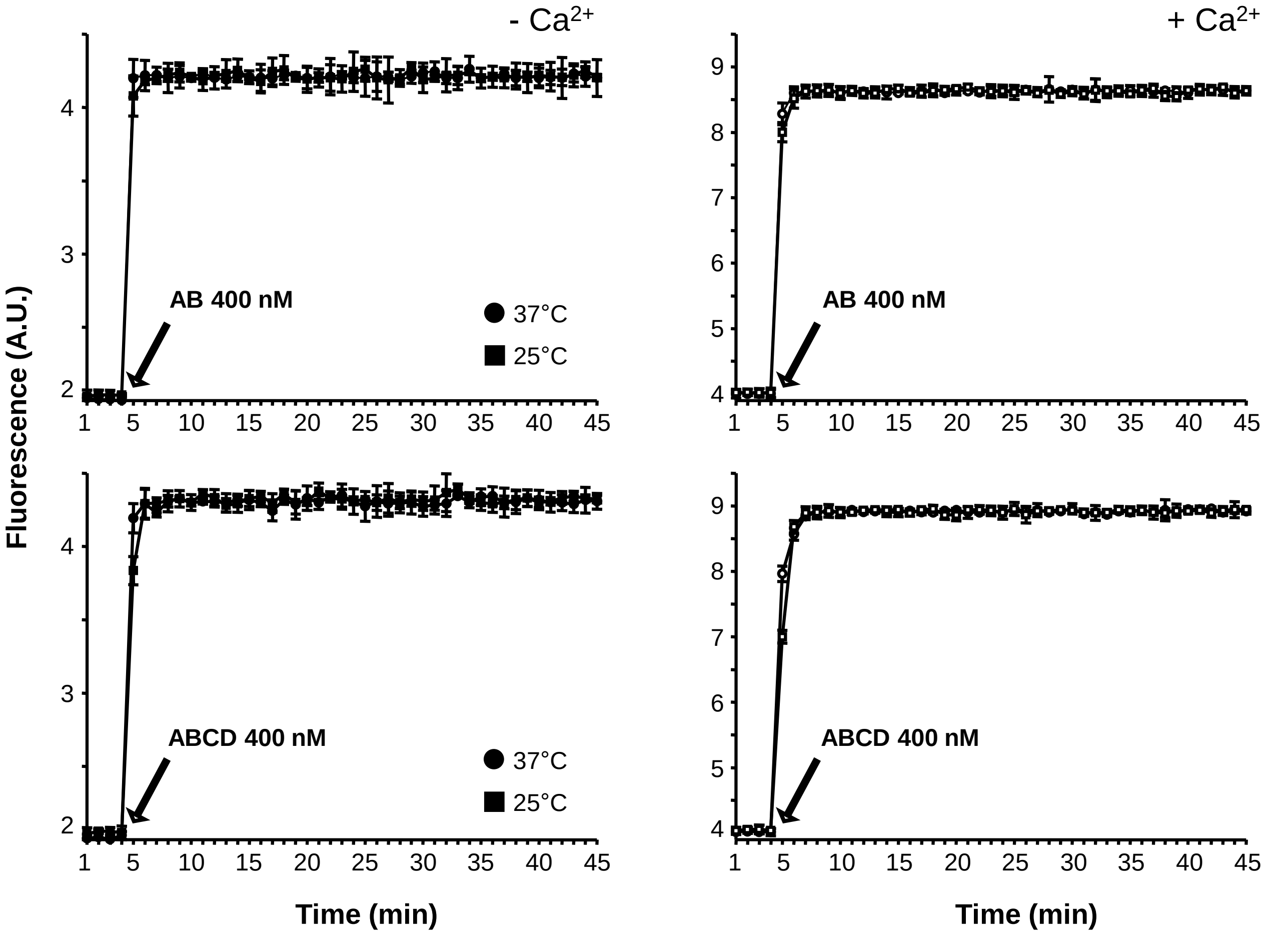

Suppl. Fig. 7. Time-course of the increase in 12.5 nM D-CaM fluorescence emission at 37 ºC and 25 ºC upon addition of GST-AB (top panels) and GST-ABCD (bottom panels) in the presence or absence (10 mM EGTA, left panels) of Ca^{2+} (3.9 µM free Ca^{2+}, right panels). Each point is the average of 4 independent experiments.

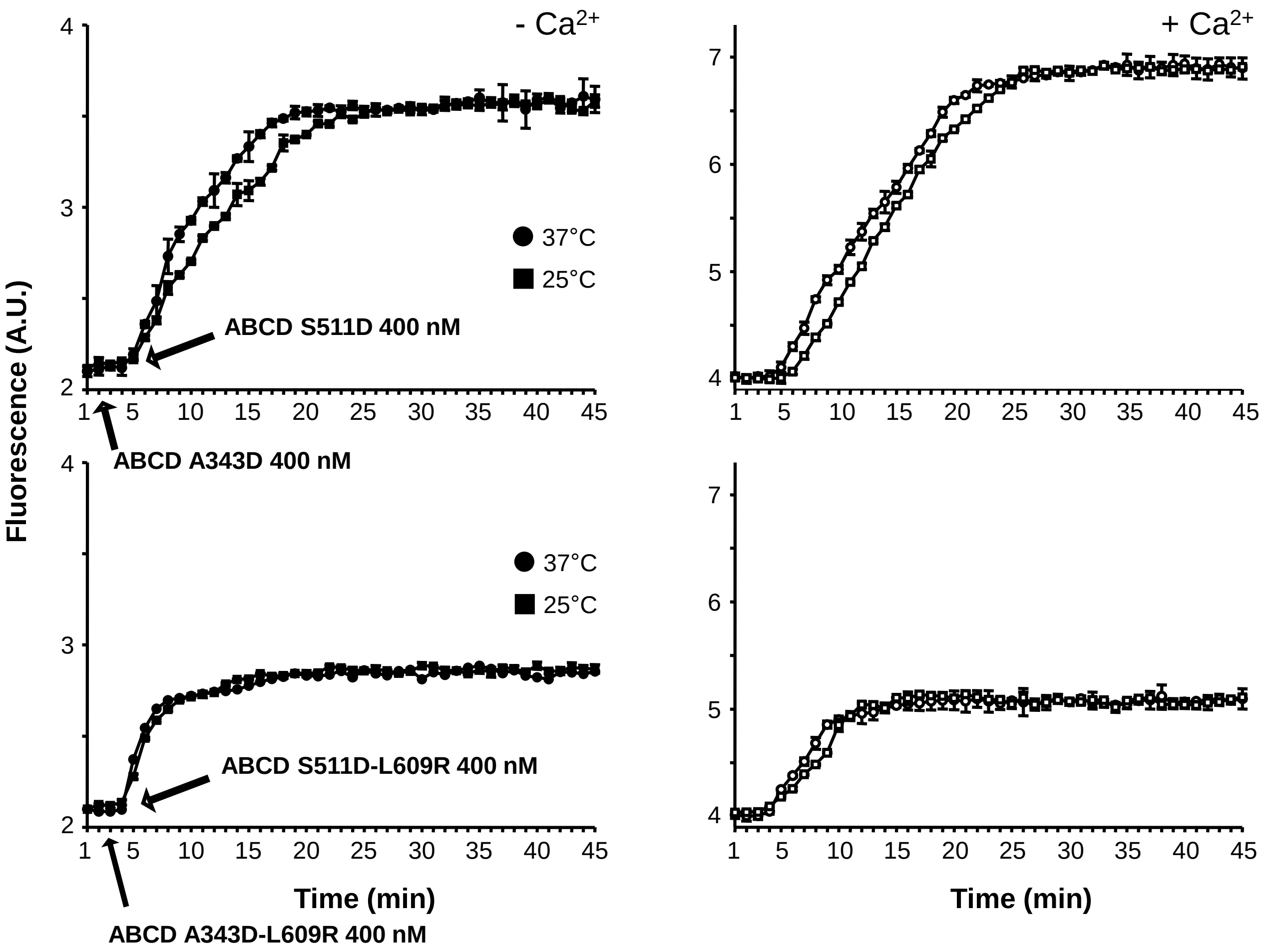

Suppl. Fig. 8. Time-course of the increase in 12.5 nM D-CaM fluorescence emission at 37 ºC and 25 ºC in the complementation assay. Each point is the average of 3 independent experiments.

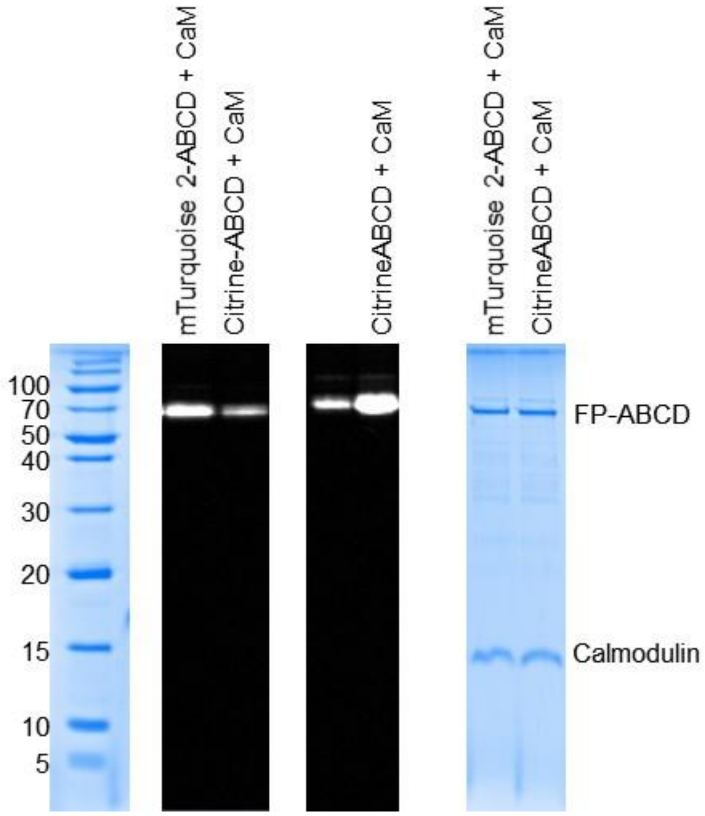

Suppl. Fig. 9. SDS-PAGE analysis of the purified FP-ABCD/Calmodulin complexes employed in this study (15% gel). Samples were not boiled to preserve the fluorescence of the FPs: first column, molecular weight markers; second column, gel fluorescence after excitation with a blue led; third column, fluorescence after excitation with a green led; fourth column, Coomassie blue staining.

Suppl. Table 1. Binding parameters obtained using D-CaM (12.5 nM) after fitting a Hill equation to the data

	- Ca^{2+} (EGTA 10 mM)			+ Ca^{2+} (3.9 μM)		
Protein	Maximal increase in fluorescence	EC_{50} (nM)	h (n)	Maximal increase in fluorescence	EC_{50} (nM)	h (n)
**AB**	111.0 ± 1.4	11.0 ± 0.5	1.6 ± 0.3 (6)	115.0 ± 2.1	27.1 ± 1.2	3.4 ± 0.5 (5)
**ABCD**	116.0 ± 0.3	9.1 ± 0.1	1.9 ± 0.1 (3)	118.0 ± 1.5	15.1 ± 0.6	2.5 ± 0.2 (3)
**ABCD S511D**	4.8 ± 0.2	N.D.	N.D. (3)	6.8 ± 0.2	N.D.	N.D. (3)
**ABCD A343D**	3.7 ± 0.2	N.D.	N.D. (3)	4.8 ± 0.3	N.D.	N.D. (3)
**ABCD L609R**	106.0 ± 1.7	30.8 ± 1.3	2.2 ± 0.2 (3)	110.0 ± 1.9	59.5 ± 2.1	2.8 ± 0.2 (3)
(ABCD S511D + ABCD A343D) **ABCD#**	76.0 ± 2.4	45.5 ± 3.8	1.5 ± 0.2 (3)	79.0 ± 3.4	78.5 ± 8.5	1.3 ± 0.1 (3)
**ABCD S511D-L609R**	4.3 ± 0.7	N.D.	N.D. (3)	4.8 ± 0.5	N.D.	N.D. (3)
**ABCD A343D-L609R**	5.1 ± 1.1	N.D.	N.D. (3)	4.3 ± 0.3	N.D.	N.D. (3)
(ABCD S511D-L609R + ABCD A343D-L609R) **ABCD#-L609R**	32.0 ± 1.1	119.0 ± 8.1	2.4 ± 0.3 (3)	28.0 ± 0.6	173.0 ± 6.2	2.8 ± 0.2 (3)
**AB S511D**	2.8 ± 0.3	N.D.	N.D. (4)	1.8 ± 0.2	N.D.	N.D. (4)
**AB A343D**	5.3 ± 0.3	N.D.	N.D. (4)	4.8 ± 0.3	N.D.	N.D. (4)
(AB S511D + AB A343D) **(AB#)**	33.0 ± 1.5	8.5 ± 1.1	1.9 ± 0.5 (4)	17 ± 1.1	22.9 ± 2.9	2.5 ± 0.7 (4)
**AB Δ2**	6.2 ± 0.4	38.2 ± 7.5	1.6 ± 0.4 (3)	6.3 ± 0.1	30.2 ± 2.7	1.6 ± 0.2 (3)
**AB Δ2-Tet**	88.0 ± 3.3	21.5 ± 0.5	2.1 ± 0.3 (3)	99.0 ± 5.9	41.4 ± 5.6	1.7 ± 0.3 (3)
**ABΔ2CD**	103.0 ± 1.9	17.8 ± 1.1	1.9 ± 0.2 (3)	101.0 ± 2.8	30.1 ± 2.2	2.4 ± 0.4 (3)

h, Hill coefficient; n, number of independent experiments; EC_{50}, concentration producing 50% of the maximal effect; n, number of independent experiments; N.D., not determined.

Please note that due to depletion of the free ligand, the EC_{50} is larger than the Kd and the values of the Hill coefficients are distorted. The interpretation of these coefficients is not straightforward.

Data for AB, AB S511D, AB A343D from[2], and data for ABCD and ABCD-L609R from[3].

Reference List

1. Sun, J. & MacKinnon, R. Cryo-EM Structure of a KCNQ1/CaM Complex Reveals Insights into Congenital Long QT Syndrome. *Cell* **169**, 1042-1050 (2017).

2. Alaimo, A. *et al.* Cooperativity between calmodulin-binding sites in Kv7.2 channels. *J. Cell Sci.* **126**, 244-253 (2013).

3. Alberdi, A. *et al.* Uncoupling PIP_2-calmodulin regulation of Kv7.2 channels by an assembly de-stabilizing epileptogenic mutation. *J. Cell Sci.* **128**, 4014-4023 (2015).

4. Alaimo, A. *et al.* Pivoting between Calmodulin Lobes Triggered by Calcium in the Kv7.2/Calmodulin Complex. *PLoS. ONE.* **9**, e86711 (2014).

5. Sachyani, D. *et al.* Structural Basis of a Kv7.1 Potassium Channel Gating Module: Studies of the Intracellular C-Terminal Domain in Complex with Calmodulin. *Structure.* **22**, 1582-1594 (2014).

6. Strulovich, R., Tobelaim, W. S., Attali, B. & Hirsch, J. A. Structural Insights into the M-Channel Proximal C-Terminus/Calmodulin Complex. *Biochemistry* **55**, 5353-5365 (2016).
